# Assessing short evolution brucellosis in a highly brucella endemic cattle keeping population of Western Uganda: a complementary use of Rose Bengal test and IgM rapid diagnostic test

**DOI:** 10.1186/s12889-018-5228-9

**Published:** 2018-03-05

**Authors:** Arnold Ezama, Jean-Paul Gonzalez, Samuel Majalija, Francis Bajunirwe

**Affiliations:** 1 Department of Production and Marketing, Office of the District Veterinary Officer, Sheema District Local Government, P.O. Box 160, Kabwohe, Sheema Uganda; 2 Center of Excellence for Emerging and Zoonotic Animal Disease (CEEZAD), Kansas State University, Office Park, 1800 Kimball Ave, Suite 130, Manhattan, Kansas 66502 United States of America; 3 Department of Biosecurity, Ecosystem and Veterinary Public Health, College of Veterinary Medicine, Animal Resources and Biosecurity, Makerere University, 7062 Kampala, Uganda; 4 Department of Community Health, Faculty of Medicine, Mbarara University of Science and Technology, P.O. Box 1410, Mbarara, Uganda

**Keywords:** *Brucella*, Cattle, Keepers, Uganda, Lateral, Flow, Assay, Rose, Bengal, Test

## Abstract

**Background:**

Brucellosis is a worldwide and zoonotic disease often sadly misdiagnosed in endemic areas. Challenges of availability and accessibility of diagnostic tools are common in resource constrained populations where the most vulnerable are found, surveillance and diagnosis are limited too.

**Methods:**

A cross-sectional study using a simple two stage cluster sampling method was conducted to measure short evolution brucellosis burden among cattle keeping households that are one of the highest risk populations to be exposed to *Brucella* infection. A total of 216 households were randomly selected from 18 rural villages from the Western Region of Uganda. Household blood samples were tested for *Brucella* antibodies using the highly sensitive Rose Bengal test (RBT) and IgM ELISA Lateral Flow Assay (LFA).

**Results:**

Among the total tested population, 58.8% did not react with any of the tests, 13.4% reacted with both tests. Among those that reacted with both (*N* = 29), 62.1% had weak (+ 1) LFA staining, 34.5% had moderate (2+) LFA staining. Altogether, both weak and moderate staining (96.5%) are consistent with sub-acute disease, while only one (3.4%) had strong (3+) LFA staining consistent with acute infection. 19.4% of the samples tested positive only with RBT, consistent with chronic infection, eighteen samples (8.3%) reacted exclusively with IgM LFA.

**Conclusion:**

We identified a high prevalence of short evolution brucellosis in the cattle keeping household members. Prevalence of chronic infection diagnosed with RBT only was higher than the prevalence of short evolution brucellosis. IgM LFA results depict possible cases of cross reaction with Salmonella spp., Plasmodium etc. Ultimately, we identified a consistent prevalence of short evolution brucellosis in the cattle keeping household members. Indeed, the use of a combined diagnostic with LFA and RBT is easy and amenable for an active disease surveillance and accurate diagnosis in rural settings.

## Background

Brucellosis is one of the most common zoonotic infections [1] and the magnitude is variable from areas with incidences ranging from greater than 500 people/Million/year, to being none existent in other regions. Worldwide overall 500,000 new cases occur per year with incidence significantly lower in developed countries [2].

Humans get infected through consumption of infective animal products and through contact with infected animal tissues like hides, placentas and aborted fetuses [3, 4]. Brucellosis is a debilitating illness, with flu like symptoms like relapsing fever, sweating, fatigue and weight loss, headache, and joint pain persisting for weeks to months. Focal lesions appear with abscess formation in various body organs including testicles and bones among others [4]. Importantly, brucellosis causes serious economic loses because of time lost by patients from daily activities [4] and losses in animal production [5].

The most recent prevalence in south western Uganda indicated as 11% among humans, 14% among the cattle, 29% in bovine milk and 17% in goat serum [6]. In developing countries misdiagnosis of Brucellosis is common majorly because there is lack of access to proper testing of Brucellosis in health centers especially in the rural settings, and also due to the none specific clinical presentation of brucellosis mimicking many other infectious disease particularly in sub-Saharan Africa (e.g. Malaria, Typhoid, viral fevers) [6]. Brucellosis is often misdiagnosed as Malaria by both self medicating patients and clinicians who only base on presumptive diagnosis, and this leads to wrong treatment using anti-plasmodial medication [7–9]. From previous studies, surveillance is an important component of brucellosis disease control [10] and requires resources that may not be readily available in sub Saharan Africa. Easy to use diagnostic equipment that are accessible to the vulnerable communities have been recommended by the World Health Organisation, and one of the essential characteristics of the diagnostic tests being single contact testing [6, 11–13]. For a rural setting, the one-time combined testing using RBT, ELISA IgM and IgG rapid diagnostic tests would be favorable for disease confirmation. The IgM and IgG LFA are a simplified version of *Brucella*-specific ELISA, and meet many of the World Health Organisation’s criteria and they have also been used before in Brucellosis disease surveillance in Uganda [6, 11, 13].

Ugandans are dependent on agriculture both for commercial and subsistence so keeping livestock is a key component of agricultural activities in Uganda [14]. However, these extended close interactions with animals expose the human population to *Brucella.*

The currently recommended Rose Bengal test (RBT) for health establishments in rural setting is noticeably absent in the health center IIs (i.e. village based government health facility offering primary care) and also absent in the Health Center iv (i.e. sub-district based-equipped facility with basic surgery and laboratory services) [15, 16]. Ranking of government health facilities in descending order of being well equipped, availability of most health services and ability to tackle major cases is as follows: hospital, Health Center (HC) iv, HC iii and HC ii [16]. Sheema district has one hospital-Kitagata hospital, two HCs iv, ten HCs iii and fifteen HCs ii, and out of these government health centers, only one health center iii facility (^1^/_28_) screened for *Brucella* infection at no cost to the patient (see Table 1). Most of the government health centers rarely do Brucellosis tests according to the information from the district veterinary and district health offices of western districts of Uganda.

**Table 1:** Number of patients that tested positive for brucellosis at the Health Center facilities of Sheema district as captured from the Health Management Information System for period January 2014 to February 2015, Uganda

Health center	Location	Catchment	Number (%)
Kabwohe Clinical Research Center (P)	Kabwohe-Itendero T/C	Sheema district	8 (13.79)
Hope Medical Center (P)	Bugongi T/C	Bugongi S/C	10 (17.24)
Rukondo health center ii (MOH)	Kasaana S/C	Rukondo village	26 (44.82)
Bugongi health center iii (MOH)	Bugongi T/C	Bugongi T/C	10 (17.24)
Rushozi Health Center ii (MOH)	Kyangyenyi	Rushozi village	4 (6.90)
Total			58 (100)

Legend: *P* Private, *MOH* Ministry of Health, *T/C* Town Council, *S/C* Sub County

The objective of the study was to find out the prevalence of short evolution (i.e. acute and sub-acute) brucellosis [17] in the cattle keeping population based on the lack or limited diagnostic tools in the government health centers and on the basis, that when private health centers screen, they use only RBT and no disease confirmation is done. The justification for this study was that getting population based data on prevalence of acute or sub-acute brucellosis infection and showing disease burden will promote the use of combination diagnostic tools of LFA and RBT to confirm disease. Using RBT only would necessitate demonstrating increasing titers from sera from patients. This demonstration means sera is tested and then more sera is got from the patient after 14 days and tested to show increase in titers, this may be difficult for patients and most rural health centers to carry out.

## Methods

A cross-sectional study using a two stage cluster sampling method [18] was carried out in Kyangyenyi Sub County, Sheema district, Uganda Western Region. Population sample size for Kyangyenyi Sub County (31,263 inhabitants) was obtained at 95% confidence interval with expected prevalence of 11% [6, 19]. From each of the six study parishes of Kyangyenyi Sub County, three villages were randomly selected, and12 households were randomly selected from each village. Study villages had an average of 115 households with 4.66 persons per household [19]. A total of 216 households were visited in a period of one month. Household eligibility was based on having at least one female bovine (i.e. associated risk with milk and pregnancy) [6, 20–22]. Household members were eligible if they satisfied at least one of the following criteria: lived together under same roof for more than a week, shared meals from a common cooking pot, took care of the cattle, carried out milking and preparing animal products for consumption [22, 23]. The village health team members listed all the households that fit the inclusion criteria and then households were randomly chosen from these lists. Eligible members of each randomly chosen household were enumerated and one member randomly chosen, if the person rescinded, another raffle without replacement was done to select another person from the household sampling frame.

Blood samples were collected in the households of the study participants according to the guidelines from the Clinical and Laboratory Standards Institute (i.e. National Committee for Clinical Laboratory Standards; Procedures for the Collection of Diagnostic Blood Specimens by venipuncture. Approved Standard - Fifth Edition H3-A5, Vol.23 No.32.). 5 μl of blood were used at point of care testing using the Test-it™ *Brucella* IgM ELISA lateral flow assay kit (LFA) (Lifeassay Diagnostics Ltd., South Africa) for the detection of IgM antibodies following the manufacturer’s instructions [13]. The remainder of the blood was kept in sterile dry vacutainer tubes (Becton Dickinson®, Plymouth, U.K.), labeled and allowed to clot for 30 min in the field, and then serum was harvested into cryogenic vials and kept at -4 °C for less than 24 h and then transported to Mbarara western regional veterinary laboratory where it was stored in liquid nitrogen and then processed within 72 h. Serum samples were screened for anti-*Brucella spp.* antibodies by agglutination using rapid slide-type agglutination assay Rose Bengal test (RBT) performed with a pinkly stained *B. abortus* suspension at pH 3.6 to 3.7, reacting samples (i.e. agglutination) were considered positive. RBT protocol for incubation time was adjusted from four to eight minutes considering that sera with blocking IgA or with high titers of non-agglutinating antibodies will need up to eight minutes to develop the bacterial clumps or the characteristic rim of positive RBT test [17, 24].

Demographic data and the results of the IgM LFA and RBT reaction were collected from each participant per household.

The study was reviewed and approved by the Committee of faculty of Medicine and the Research Ethics Committee of Mbarara University of Science and Technology (MUIRC 1/7) (06/06, 2016 / Study #160121).

## Results

The participants were predominantly male 61% (131) with a mean age in years of 49 (±17.7 SD). A total of 216 individuals were tested using IgM *Brucella* (LFA) and the Rose-Bengal test (RBT), among them 127 (58.8%) did not react with any of the tests, 29 (13.4%) reacted with both tests. Among these double reacting, 18/29 (62.1%) had weak (1+) LFA staining, 10/29 (34.5%) had moderate (2+) LFA staining, both weak and moderate staining are considered consistent with sub-acute disease 28/29 (96.5%) and only one (3.4%) had strong (3+) LFA staining consistent with acute infection [17, 25]. Among the total tested, 42 (19.4%) reacted only with RBT which is consistent with probable case definition for *Brucella* infection, and, 18 (8.3%) reacted only with IgM LFA (See Table 2).

**Table 2:** Complementary screening results of study participants against brucellosis using *Brucella* IgM Lateral Flow Assay and the Rose Bengal Test in cattle keepers (Kyangyenyi sub-county, Sheema district, Uganda, 2016)

Rapid IgM Test	Rose Bengal Test		
	Negative	Positive 4 min	Positive 8 min only
Negative	127	23	19
Positve1+	16	13	5
Positive2+	2	10	0
Positive 3+	0	1	0

## Discussion

The thrust of the study was to find out the prevalence of short term evolution brucellosis in members of cattle keeping households using recommended criteria for disease confirmation [17, 26, 27]. Since other complementary tests are not amenable and not easily available in low income settings, the study depicted the ease of using LFA in disease surveillance and diagnosis in rural settings.

Since this was an epidemiological cross-sectional study where participants were chosen on parameters that would expose them to *Brucella* infection and not on clinical grounds, it is not surprising that 58.8% of the study participants had negative results from both tests. Just like in the study by Irmak et al. [25], there were no additional clinical cases from the community survey. That community based survey had been a follow up strategy since the hospital had recorded significant cases at that time. While we expected some form of exposure from this known endemic area [3, 6], we identified only one acute case of Brucellosis (3.4%) who reacted with both IgM LFA and RBT. Among the confirmed short evolution cases, 96.5% of the cases were sub-acute which was an anticipated proportion by nature of the study design in such an endemic population, where we expected to find more sub-acute cases tending towards chronic [28], than acute or newly infected cases. The overall prevalence of short evolution brucellosis was 13.4% which also explains the fact that in a highly exposed population, like cattle keeping households, we expect significant levels of short evolution brucellosis given the 29% prevalence in livestock in this area [6].

According to WHO, using only RBT in an endemic population, positive results can be taken as a probable case [6, 26, 27]. In our study,19.4% were chronic *Brucella* infection only detected by RBT, corresponding to cases with past infection or long evolution disease producing majorly IgG and IgA with low or un detectable levels of IgM [13, 17, 29] and in accordance with the status of a general population leaving in an endemic area.

As per the CDC and WHO guidelines, it is advisable to use two tests, agglutinating and non- agglutinating, to confirm disease [26, 29, 30], the IgM LFA was able to detect 18 participants as reactive without any reaction with RBT. We cannot rule out the potential of false positives probably from cross-reacting IgM with Plasmodium, *Escherichia coli O157*, *Francisella tularensis*, *Yersinia enterocolitica*, *Vibrio cholerae* and *Salmonella* species [29, 31]. With increase in Malaria prevalence in Uganda [32] where 95% of the country is endemic with Malaria [33] and more than 60% of rural dwellers do not treat their water before drinking, this depicts the high propensity for typhoid infection and carrier state in these populations [34], it is highly possible we had cases of cross reactivity because RBT is highly sensitive in acute cases of brucellosis [17, 35] making it unlikely that 18 cases would be undetectable by RBT.

## Conclusions

The prevalence of short evolution brucellosis in the cattle keeping household members (13.4%) increased with an increment of 2.4 as compared to previous brucellosis prevalence of 11% depicted in this region in a study done earlier in 2015 [6]. Prevalence of chronic infection at the level of probable case definition, diagnosed with RBT only was higher than the prevalence of short evolution brucellosis. IgM LFA results only, depict possibly cases of cross reaction, so the results from IgM LFA in Brucellosis, Typhoid and Malaria endemic populations should be confirmed with other agglutinating tests such as RBT.

The strength of the study was based on the ability to use combination of agglutinating and non-agglutinating tests and, on the low-cost and an easy to use diagnostic by RBT and LFA to confirm short evolution brucellosis in members of cattle keeping households in a brucellosis endemic area. Considering the present results, we recommend that febrile conditions must also be tested for Brucellosis when tested for Malaria (or other febrile diseases). Cattle vaccination needs to be encouraged in cattle keeping communities and continuous surveillance of brucellosis be done for both humans and animals. Future studies would gain from carrying out a societal cost-effective modeling of introduction of LFA with the already available Malaria RDT kits to village health team members in *Brucella* endemic human populations.

## Abbreviations

ELISA: Enzyme linked immuno-sorbent assay
H/C: Health center
H/H: House Hold
HMIS: Health management information system
IgA: Immune globulin A
IgG: Immunoglobulin G
IgM: Immunoglobulin M
LFA: Lateral Flow Assay
MAAIF: Ministry of agriculture animal industry and fisheries
MUIRC: Mbarara University Institutional Review Committee
RBT: Rose Bengal test
*Spp*: Species
U.K: United Kingdom
U.S.A: United States of America
W.H.O: World Health Organization
